# Monoterpenes from Larval Frass of Two Cerambycids as Chemical Cues for a Parasitoid, *Dastarcus helophoroides*

**DOI:** 10.1673/031.013.5901

**Published:** 2013-06-21

**Authors:** Jian-Rong Wei, Xi-Ping Lu, Li Jiang

**Affiliations:** 1 College of Life Sciences, Hebei University, Hebei Baoding, 071002, China; 2 College of Plant Protection, Shandong Agricultural University, Shandong Taian, 271018, China; 3 Taian Forestry Bureau, Shandong Taian, 271000, China

**Keywords:** kairomone, olfactory response, parasitoid, tritrophic interactions, woodborer

## Abstract

*Anopiophora glabripennis* (Motsch.) (Coleoptera: Cerambycidae) is a destructive woodborer, attacking many species of deciduous hardwood trees. *Apriona swainsoni* (Hope) (Coleoptera: Cerambycidae) is a woodborer of *Sophora japonica* L. (Angiospermae: Fabaceae). *Dastarcus helophoroides* (Fairmaire) (Coleoptera: Bothrideridae) is an important natural enemy of both Cerambycid species in China. Kairomones for two populations of *D. helophoroides* that parasitize *A. glabripennis* and *A. swainsoni* respectively were studied. Based on identification and quantification of volatiles from larval frass produced by *A. glabripennis* and *A. swainsoni*, monoterpenes were selected to test their kairomonal activity to both populations of *D. helophoroides* adults using a Y-tube olfactometer. The results indicated that (*S*)-(-)-limonene served as a kairomone for the population of *D. helophoroides* parasitized *A. glabripennis*. α-pinene, (*IR*)-(+)-αpinene and (+)-β-pinene were attractive to the population of *D. helophoroides* parasitized *A. swainsoni*. The results provide information about the co-evolution of *D. helophoroides*, its host, and host-food trees.

## Introduction

*Apriona swainsoni* (Hope) (Coleoptera: Cerambycidae) is a woodborer and a major threat to the historical and famous tree *Sophora japonica* L. (Angiospermae: Fabaceae), which is the civic tree in many cities in China (Tang and Liu 2000). *Anoplophora glabripennis* (Motsch.) (Coleoptera: Cerambycidae) is a serious wood-boring pest that infests many broad-leaved tree species in northern China (Gao and Li 2001; Hu et al. 2009). Both pests attack healthy trees and spend most of their life as larvae boring inside tree trunks and large branches, eventually causing mortality. In the late 1990s, *A. glabripennis* was discovered as an invasive pest in the United States, which prompted a major eradication effort (Nowak et al. 2001; Macleod et al. 2002; Hu et al. 2009). *A. swainsoni* is listed as a quarantine forest insect pest in some provinces in China (Tang and Liu 2000).

*Dastarcus helophoroides* (Fairmaire) (Coleoptera: Bothrideridae) is an important ectoparasitoid of Cerambycid beetles and is distributed in most provinces of China (Qin and Gao 1988; Wang et al. 1996; Wei et al. 2009a), some areas of Japan (Tadahisa 2003), and Korea (Lim et al. 2012). Studies on this species have been conducted investigating its biology (Zhou et al. 1985; Qin and Gao 1988; Lei et al. 2003), behavior (Wei et al. 2008a), and mass-rearing techniques (Ogura et al. 1999; Wang et al. 1999; Shang et al. 2009). Adults are polyphagous, mainly feeding on corpses of other insects (Qin and Gao 1988); sometimes, they also attack live larvae of longhorned beetles (Wei et al. 2008a). During most of their lifetime, they stay motionless under the bark crevice or in the caves of trees. Eggs are laid on the outer surface of the bark near the host entrance hole, frass-extrusion hole, or around the host larvae tunnel walls (Qin and Gao 1988). Once hatched, first instar larvae have legs and can actively move to locate hosts, but their legs degenerate once parasitism has occurred. They pupate next to the host corpse. *D. helophoroides* lay eggs twice a year in the field (Qin and Gao 1988), but in the laboratory, females can lay eggs at 4 to 6 months intervals at 20–27° C regardless of the presence of a suitable host (Wei and Jiang 2011). The egg, larval, and pupal periods are respectively 12.7, 8.4, 25.6 days on average at 21 ± 1° C (Lei et al. 2003). Most adults can live over 3 years (Wei et al. 2007). They are tolerant of starvation and can live over 60 days without food or water (Wei et al. 2009a). Since the 1980s, *D. helophoroides* has been used as a biological agent to control *A. glabripennis, Batocera horsfieldi* (Hope), and *Massicus raddei* (Blessig) in China, and *Monochamus alternates* Hope in Japan (Zhou et al. 1985; Qin and Gao 1988; Tadahisa 2003; Li et al. 2009; Wei et al. 2009b).

Some parasitoids and predators utilize specific chemical cues from the firass of herbivores as a host-finding strategy (Grégoire et al. 1991; Sullivan et al. 2000; Pettersson 2001; Chuche et al. 2006). There are three different populations of *D. helophoroides* in China that differ in their olfactory response to frass of different longhorned beetle species (Wei et al. 2009c). One population of *D. helophoroides* that parasitized larvae and pupae of *A. glabripennis* was attracted to the host larval frass from *Salix babylonica* L. (Malpighiales: Salicaceae) (Wei and Jiang 2011). Recent studies showed that a population of *D. helophoroides* that was collected on *S. japonica* was attracted to the larval frass of *A. swainsoni* (Lu et al. 2012). Wei et al. (2008b) reported that one of the populations of *D. helophoroides* that parasitizes *M. raddei* (Blessig) on *Quercusmongolicus* Fisch, ex. Turcz in northern China uses larval frass of *M. raddei* as a major cue while searching for hosts, and the chiral monoterpene (*R*)-(+)-limonene is a kairomone of *M. raddei* for this population of *D. helophoroides.* Larval frass of *A. glabripennis* and *A. swainsoni* both release monoterpenes (Ding et al. 2009; Jiang et al. 2010). Therefore, we put forward a hypothesis that different populations of *D. helophoroides* might use similar monoterpenes as their kairomones when searching for their hosts.

The objectives of this study were to analyze monoterpenes and stereoisomers of chemicals released both from larval frass of *A. glabripennis* feeding on *S. babylonica* and larval frass of *A. swainsoni* feeding on *S. japonica*, and to test selected monoterpenes and their isomers against adults of related *D. helophoroides* populations to identify the unique kairomone that may aid *D. helophoroides* adults in searching for their hosts. The information gained will further the understanding of the co-evolution of this parasitoid, its host, and host tree, and will benefit the development and improvement of effective and sustainable biological control methods for two Cerambycid species management.

## Materials and Methods

### Insects

Adults of type G *D. helophoroides* were from a laboratory colony established from parasitized larvae and pupae of *A. glabripennis* collected from trunks of *S. babylonica* in Xian, Shaanxi province, China (latitude 34° 15′ N, longitude 108° 50′ E, altitude 450 m a.s.l.) in April 2006. Adults of type S *D. helophoroides* were from a laboratory colony established from parasitized larvae and pupae of *A. swainsoni* collected from trunks of *S. japonica* in Qufu city (latitude 35° 39′ N, lon-gitude 116° 98′ E, altitude 60 m a.s.l.) and Feicheng city (latitude 36° 24′ N, longitude 116° 76′ E, altitude 57 m a.s.l.), Shandong Province, China, in February 2006. Both colonies have been maintained at the Research Institute of Forest Ecology, Environment and Protection, Chinese Academy of Forestry, Beijing, China. Larvae were reared on a substitute host (*Thyestilla gebleri* (Fald.) (Coleoptera: Cerambycidae)), and adults were reared with corpse powder of locust and *Tenebrio molitor* L. (Coleoptera: Tenebrionide). All tested adults were fifth generation offspring and 3–12 months old.

### Host frass field collection

Larval frass of *A. glabripennis* was collected from *S. babylonica* in Xian during May 2008. Larval frass of *A. swainsoni* was collected from *S. japonica* in Taian city, Shandong province (latitude 35° 50′ N, longitude 117° 05′ E, altitude 150 m a.s.l.) during June 2008. Frass was collected from larvae tunneling in galleries by cutting off the outer bark of tree trunks and scooping frass into vials. Frass (about 120 g) from more than 30 heavily damaged trees was collected and mixed together in capped, clean glass bottles and kept at -20° C in the laboratory until tested.

### Monoterpenes collection and quantification

Samples of *A. glabripennis* larval frass (15 g) and *A. swainsoni* larval frass (15 g) were aerated separately in a clean, glass conical flask (300 mL) with an air entrance (inner diameter: 0.3 cm) at the bottom and a vent (inner diameter: 0.3 cm) at the top (diameter: 2.5 cm). The vent was connected by 10 cm Teflon tubing to a Porapak Q trap (200 mg in a glass tube, 80–100 mesh; Waters Associates Inc., www.waters.com) (Wei et al. 2008b). Pure nitrogen (99.999%), warmed to 26° C by passing through an electric thermostat, was directed through the bottom entrance of the flask and subsequently purged headspace volatiles into the trap that was connected to the vent on the top of the flask with a Teflon tube. For each frass sample, there were three replicates. Before samples were run, each clean, empty conical flask was aerated using heated nitrogen (about 26° C) for 10 min to reduce potential contamination. The flow rate of nitrogen was 100 mL/min, and the samples were aerated for 4 hours. Volatiles trapped in Porapak Q were rinsed with 200 µl dichloromethane, and 2 µg of internal standard (heptyl acetate) was added for quantification analysis.

The configuration and quantification of monoterpenes in the extracts were analyzed by gas chromatographic enantiomer separation on a chiral column (Cyclosil-B, 30 m × 0.25 mm × 0.25 µm; Agilent, www.agilent.com) equipped on an Agilent 7890 GC with an FID detector. The carrier gas was pure nitrogen, and the injector temperature was 220° C. The column temperature was programmed at 45° C for 1 min, increased at 1.5° C/min to 72° C, held for 6 min, then increased at 6° C/min to 120° C and 10° C/min to 220° C, and held for 10 min. Injections of 1 µl of each extract were made. The different synthetic monoterpenes indentified (Ding et al. 2009; Jiang et al. 2010) were also injected to compare their retention times with peaks in extracts. Quantification of each compound in the extracts was determined by comparing to the internal standard.

### Olfactory responses of *Dastarcus helophoroides* adult to synthetic monoterpenes

All commercially available monoterpenes and their isomers identified in frass were evaluated to identify behaviorally active components that may serve as kairomones for type G and type S *D. helophoroides*. The orientational responses of *D. helophoroides* adults to different odor sources were tested using a Y-tube olfactometer. The bioassay setting and procedure were as described in Wei et al. (2008b; 2011). The beetles were challenged with synthetic compounds in an odor dispenser at one arm and a blank control at the other arm of the Y-tube olfactometer. A special odor dispenser was designed to control the amount of chemical released. It consisted of a polymerase chain reaction tube (0.5 mL, Eppendorf, www.eppendorf.com) containing 10 µl of each undiluted synthetic compound, with two large holes (diameter: 0.49 ± 0.10 mm) and two small holes (diameter: 0.22 ± 0.05 mm) made by puncturing the wall with a needle, that was inserted into a centrifuge tube (2.0 mL, Eppendorf, Corp.) with two holes (diameter: 1.11 ± 0.02 mm) made by puncturing the wall with a needle. Both the polymerase chain reaction tube and the centrifuge tube were capped tightly (Wei et al. 2008b). Dispensers were replaced with fresh ones every day to ensure consistent release rate throughout the entire experiment. The average release rates of different monoterpenes were 20–30 µg/hr by weighed odor dispensers each day.

Insects were introduced into the release chamber individually. Observations were taken 30 min after introduction to note whether the insect had made a choice (traveled at least to the midpoint of one of the arms or already dropped into one of the glass containers). Beetles that did not respond within 30 min (stayed in the release chamber or did not reach to the midpoint of the arms) were excluded from data analysis. Individual beetles were tested only once. At least 30 adults should respond per treatment for statistical analysis. Connections between the odor source containers and olfactometer arms were exchanged after every 5 beetles tested in order to exclude directional bias in beetle choices. After each experiment, the olfactometer was disconnected from the containers and thoroughly washed with a detergent, rinsed in 70% ethanol, and dried by heated dry air. Experiments were conducted at 23–26° C and 60 ± 10% RH. All the bioassays were conducted from May to October in 2008 and 2009.

**Table 1.**
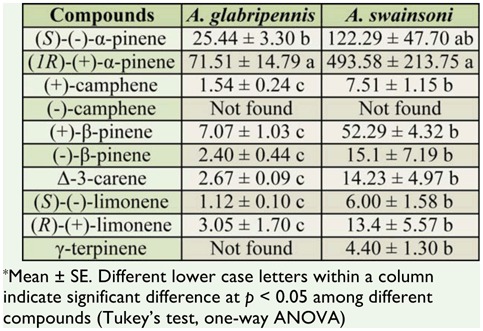
Release rate (ng/hr/g) of monoterpenes from larval frass of *Anaplophora glabripennis* or *Apriona swainsoni*.

Wei et al. (2011) showed that both sexes of *D. helophoroides* adults were attracted to frass of *A. glabripennis*. This result is important, as the sexes of *D. helophoroides* adults can only be determined reliably by dissecting the reproductive organs (Tadahisa 2003). Therefore, the insects used in this study were not sexed either before or after testing. Before being tested, the insects were starved individually in glass tubes at 25° C, 60% RH, and under 20–30 Lux (10:14 L:D) for 30–35 days to maximize their response.

### Synthetic compounds

Commercially available compounds from Sigma-Aldrich (www.sigmaaldrich.com) were used for identification and bioassay purposes were: α-pinene, 98%; (*1S*)-(-)-α-pinene, 98%, ee: ≥ 81% (GLC); (*1R*)-(+)-α-pinene, 98%, ee: ≥ 91% (GLC); camphene, 95%; (+)camphene, 80%; (-)-camphene, 75%; (-)-βpinene, 99%, ee: 97% (GLC); (+)-β-pinene, 99%; δ-3-carene, 90%; limonene, 99%; (*S*)-(-)-limonene, 96%; (*R*)-(+)-limonene, 96%, ee: 98% (GLC); γ-terpinene, 97%.

### Calculations and statistical analysis

A Wilcoxon test (Two-Related-Sample Test of Nonparametric Tests) was used to determine the differences in the responses of *D. helophoroides* adults in the olfactometer bioassays. The release rates of different compounds (i.e., the amount of a compound (ng) being released per hr per g of material) were compared with a One-Way ANOVA after square-root transformation, and was followed by multiple comparisons with Tukey's tests. All statistical analyses were carried out by SPSS software (IBM, http://www01.ibm.com/software/analytics/spss/) using a α-level of 0.05.

## Results

### Quantification of monoterpenes released from larval frass of *A. glabripennis* and *A. swainsoni*

Analysis of enantiomeric composition of volatiles trapped in Porapak Q from larval frass of *A. glabripennis* and *A. swainsoni* indicated that different isomers of some monoterpenes were present (Table 1). In most cases, the release rates (ng/hr/g) of both isomers of apinene were significantly higher than those of other compounds in the extracts of larval frass. γ-Terpine was not found in the extract sample of *A. glabripennis* larval frass. (+)-βpinene released from larval frass of *A. swainsoni* reached 52.29 ng/hr for each gram of larval frass (Table 1), though its release rate was not significantly higher than that of other monoterpenes except for two isomers of αpinene. The average release rates of monoterpenes from larval frass of *A. swainsoni* were higher than those from larval frass of *A. glabripennis* (Table 1).

### Olfactory responses of *D. helophoroides* adults to synthetic monoterpenes

**Figure 1.**
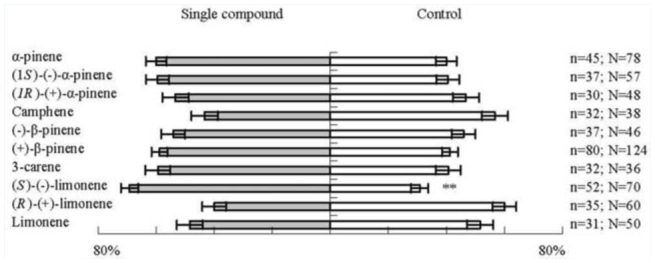
Orientational response of type G *Dastarcus helophoroides* adults to synthetic compounds in the Y-tube olfactometer. N = the number of tested adults; n = the number of adults that made a choice; bars indicate the percentages ± SE of n that chose the sample odor or control; ** = significantly different at *p* < 0.01, *Wilcoxon* test. High quality figures are available online.

A total of 11 identified monoterpenes, including different isomers, were tested for attractiveness to type G and type S adults of *D. helophoroides* in the Y-tube olfactometer. For type G *D. helophoroides*, only (*S*)-(-)limonene attracted significantly more *D. helophoroides* than the control (Z = -2.77; *p* < 0.01) (Figure 1). All of the other monoterpenes were not significantly different from the control in attractiveness to this population of *D. helophoroides*.

**Figure 2.**
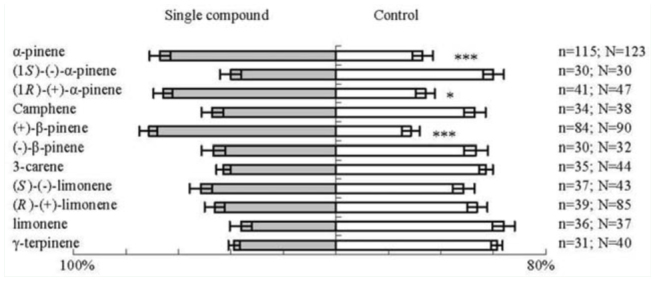
Orientational response of type S *Dastarcus helophoroides* adults to synthetic compounds in the Y-tube olfactometer. N = the number of tested adults; n = the number of adults that made a choice; bars indicate the percentages ± SE of n that chose compound odor or control; * = significantly different at *p* < 0.05; ** = significantly different at *p* < 0.001, *Wilcoxon* test. High quality figures are available online.

For type S *D. helophoroides* adults, α-pinene, (*1R*)-(+)-α-pinene, and (+)-β-pinene attracted significantly more adults than the control (Z = -3.64 , p < 0.001; Z = -2.03, *p* < 0.05; Z = 3.93, *p* < 0.001, respectively) in the Y-tube olfactometer (Figure 2). Other monoterpenes were not attractive to this population of *D. helophoroides* in the Y-tube olfactometer.

## Discussion

### Monoterpenes as kairomones of different populations of *D. helophoroides*

Most plants can release monoterpenes when attacked by insect pest (Luik et al. 1999; Chen et al. 2002; Iason et al. 2011). Many species of Coleopteran use tree terpenes as kairomones or pheromones (Byers 1992; Hobson et al. 1993; Allison et al. 2001, 2004; Thoss and Byers 2006). For instance, the volatile monoterpenes are often found serving as major attractants of Cerambycids (Allison et al. 2004), bark beetles (Byers 1992; Hobson et al. 1993; Thoss and Byers 2006), and a pine weevil (Nordlander 1990; 1991), or as major chemical cues for natural enemies searching for pine herbivores (Pettersson 2001; Mumm and Hilker 2006). The present study showed that two populations of *D. helophoroides* adults also used terpenes as kairomones to look for their potential hosts.

Some herbivores and natural enemies show chiral specificity in responses to host plant semiochemicals (Miller et al. 1996; Wibe et al. 1998; Allison et al. 2001; Erbilgin and Raffa 2001; Mozuraitis et al. 2002; Hull et al. 2004). Hobson et al. (1993) found that *Dendroctonus valens* LeConte is strongly affected by the enantiomeric composition of monoterpenes. Also, the natural enemies of *Ips pini* (Say), *Thanasimus dubius* (F.), and *Platysoma cylindrica* (Paykull) are known to prefer different optical isomers of ipsdienol (Raffa and Klepzig 1989; Raffa and Dahlsten 1995). The results of our study showed that two populations of *D. helophoroides* used different chiral monoterpenes as kairomones. This result suggests that (*S*)-(-)-limonene is an important component of the attractant kairomone for type G *D. helophoroides* adults searching for *A. glabripennis*. (*R*)-(+)-limonene and limonene were not attractive to this population, which indicates that type G *D. helophoroides* specializes on the (*S*)-(-) isomer. For type S *D. helophoroides*, adults were attracted to three monoterpenes.

### Specialization of different populations of *D. helophoroides* on kairomone

The ancestors of both type G and type S populations were collected from different ecological niches, as they parasitized different hosts on different host tree species in different places. Since this parasitoid lives in caves and is a less-active species (Wang et al 1996; Wei et al. 2008a), it is possible that they have adapted to different ecological environments and developed into different populations after long years of evolving with their own host species and host tree species (Kester and Barbosa 1991; Magalhães et al. 2007). Tree species certainly influence the frass volatiles of woodborers (Wei et al. 2011).

All tested adults from both types of populations were fifth generation. Because every generation of *D. helophoroides* was developed from the first batch of eggs laid by adults of the new generation, the fifth generation was reached after two years of rearing. The results showed that both populations retained their differences in olfactory response to chemical cues, even though both were reared from the same species of substitute host in laboratory. Therefore, both populations were specializing on the kairomones relative to their respective hosts.

Adults of type S *D. helophoroides* were attracted to more than one compound, which indicated that the type S population might be more original than other *D. helophoroides* populations, since this population is less specialized on chemical signals and can respond to more compounds, or it is a mixed population, which might respond to more Cerambycid species. Therefore, it is necessary to test the olfactory response of type S *D. helophoroides* to frass odors of different longhorned beetle species in the future.

For more distinct discrimination of different populations of *D. helophoroides*, genetic relationships of different populations need to be studied (Vaughn and Antolin 1998). Furthermore, whether the different populations are geographically distinct or overlapping needs to be clarified.

### Generalist or specialist

From the concept of species, *D. helophoroides* is referred to as a generalist or a polyphagous parasitoid because it can parasitize many Cerambycid species (Qin and Gao 1988; Wang et al. 1996). However, there exist different populations of *D. helophoroides* in nature, and they specialize on their own hosts and host tree species, so *D. helophoroides* might be referred to more accurately as a “habitat specialist” or “host specialist” (Jermy 1988; Gaston 1990; Wei et al. 2009c).

### The release rates of chemicals from larval frass and selected compounds tested in bioassay

The average release rates of monoterpenes from larval frass of *A. swainsoni* were higher than that from larval frass of *A. glabripennis* (Table 1). However, since the monoterpenes exist in almost every plant species and plant extract, and the results from the olfactory test showed that different populations of *D. helophoroides* specialize on different monoterpenes when tested using similar release rates in the laboratory, comparison of quantities of monoterpenes between larval frass from two Cerambycids might not have real meaning.

The mixtures of different monoterpenes were not tested for either *D. helophoroides* population, since the release rates of different monoterpenes from the mixture were difficult to control in order to comply with the natural release rates or ratios of different monoterpenes in the larval frass of longhorned beetles.

### Kairomone used as an attractant in the field

*D. helophoroides* is a cave beetle (Wang et al. 1996), so it is difficult to find in the field. Because the longevity of adults breeding in the laboratory decreased after several generations of rearing, it is necessary to develop some attractant to lure and trap *D. helophoroides* in the field in order to collect enough wild individuals to establish a new colony to mass rear. Based on the results in this study, kairomones can be used as attractants to lure *D. helophoroides* adults.

After (*R*)-(+)-Limonene was found as a kairomone of *M. raddei* (Wei et al. 2008b) to a population of *D. helophoroides*, (*R*)-(+)limonene was used as a lure to trap the *D. helophoroides* adults in the field of KuanDian County, Liaoning Province, China (latitude 40° 44′ N, longitude 125° 11′ E, altitude 540 m a.s.l.) in our study, where *M. raddei* caused serious damage to *Quercus liaotungensis* Koidz and adults of *D. helophoroides* could be found in the larval tunnels of *M. raddei*. However, only a few *D. helophoroides* adults were captured on average in each trap. Therefore, there could be other compounds also acting as chemical cues to attract *D. helophoroides* adults. A similar phenomenon could also be happening in two populations of *D. helophoroides* parasitizing *A. glabripennis* and *A. swainsoni*. On the other hand, the monoterpenes might be too volatile as an attractant of *D. helophoroides*, or there may be other environmental factors that also influence the behavior of insect (Hunter 2002). Further field trap experiments should be conducted based on controlled volatile ratios.
